# The therapeutic effect of adipose-derived stem cells on soft tissue injury after radiotherapy and their value for breast reconstruction

**DOI:** 10.1186/s13287-022-02952-7

**Published:** 2022-10-04

**Authors:** Haojing Tang, Yufei He, Zhuokai Liang, Jian Li, Ziqing Dong, Yunjun Liao

**Affiliations:** The Department of Plastic and Cosmetic Surgery, Nanfang Hospital, Southern Medical University, 1838 Guangzhou North Road, Guangzhou, 510515 Guangdong China

**Keywords:** Breast cancer, Breast reconstruction, Postmastectomy radiotherapy, Adipose-derived stem cells, Stem cell therapy

## Abstract

**Background:**

Postmastectomy radiotherapy is considered to be a necessary treatment in the therapy of breast cancer, while it will cause soft tissue damage and complications, which are closely related to the success rate and effectiveness of breast reconstruction. After radiotherapy, cutaneous tissue becomes thin and brittle, and its compliance decreases. Component fat grafting and adipose-derived stem cell therapy are considered to have great potential in treating radiation damage and improving skin compliance after radiotherapy.

**Main body:**

In this paper, the basic types and pathological mechanisms of skin and soft tissue damage to breast skin caused by radiation therapy are described. The 2015–2021 studies related to stem cell therapy in PubMed were also reviewed. Studies suggest that adipose-derived stem cells exert their biological effects mainly through cargoes carried in extracellular vesicles and soluble secreted factors. Compared to traditional fat graft breast reconstruction, ADSC therapy amplifies the effects of stem cells in it. In order to obtain a more purposeful therapeutic effect, proper stem cell pretreatment may achieve more ideal and safe results.

**Conclusion:**

Recent research works about ADSCs and other MSCs mainly focus on curative effects in the acute phase of radiation injury, and there is little research about treatment of chronic phase complications. The efficacy of stem cell therapy on alleviating skin fibrosis and its underlying mechanism require further research.

## Introduction

Radiotherapy plays an important role in the treatment of malignant tumors. Postmastectomy radiotherapy (PMRT) is a more commonly performed and safer option. An early comprehensive meta-analysis [1] analyzed the data of 20,000 early breast cancer patients in a breast cancer clinical trial and showed that PMRT reduced the local regional recurrence rate of lymph node-positive patients from 27% to 9% and had little effect on overall survival during the first decade or so.

However, radiotherapy inevitably causes soft tissue damage and complications, which are closely related to the success rate and effectiveness of and satisfaction with breast reconstruction [2–4]. After radiotherapy, cutaneous tissue becomes thin and brittle, and its compliance decreases. Consequently, it is often impossible to achieve good results when a dilator is simply used for reconstruction. Krueger et al. [5] reported that breast cancer patients who received radiation therapy had a higher probability of failure and complications during breast reconstruction with dilators or implants than those who did not receive radiation therapy, with failure rates of 37% (7 of 19 cases) and 8% (5 of 62 cases), respectively. Nava et al. [6] showed that the probability of capsular contraction upon breast reconstruction using implants is significantly increased if tissues are undergoing radiotherapy. In addition, PMRT has a considerable impact on the success rate of breast reconstruction after musculocutaneous flap transplantation. The complication rate with the transverse rectus abdominis myocutaneous flap, which has the best cosmetic effect after reconstruction, is as high as 63%, which is much higher than the complication rates with other reconstruction procedures using a myocutaneous flap [7, 8].

The decision of whether to perform radiotherapy as well as the sequence of radiotherapy and breast reconstruction is closely related to the recurrence of breast cancer and the quality of breast reconstruction. The sequelae of radiotherapy hinder reconstruction, and clinicians must therefore make a trade-off between safety and reconstruction quality. Therefore, research of how to prevent skin and soft tissue damage hindering breast reconstruction after radiotherapy is of great value.

In recent years, autologous fat grafting has attracted much attention based on its small residual scar and convenience. There are gradually some studies indicated that autologous fat grafting can partially improve the problem of poor skin compliance after radiation fibrosis [9–11]. ADSCs are believed to be the most important and potent fat component [12]. It is of great value to further explore the therapeutic effect and application prospect of ADSCs in radiation tissue injury for the improvement of breast reconstruction treatment methods.

## The basic biological mechanism by which radiotherapy causes cell damage

### Direct cellular damage

Radioresistance varies according to the stage of the cell cycle. Cyclin-dependent kinase inhibitors, such as P16, P21, and P53, are common checkpoint proteins expressed during the radiation-resistant phase.

In G1 phase, these inhibitors are expressed to ensure that the cell successfully enters the DNA synthesis phase (S phase). Once the cell enters S phase, these inhibitors and enzymes responsible for DNA repair are overexpressed to ensure the accuracy and integrity of DNA synthesis. Due to residual levels of S phase enzymes and cyclin-dependent kinase inhibitors, cells in G2 phase are also considered to be radiation-resistant.

In early M stage, chromosomes condense, and DNA is very susceptible to radiation damage. In general, there is a lack of repair mechanisms in M phase, and condensed and concentrated DNA is a perfect target for ionizing radiation. Therefore, M phase cells, i.e., dividing cells, are particularly radiosensitive [13].

Owing to this characteristic, cancer cells are the main target of radiotherapy. However, in addition to cancer cells, some normal cells that divide vigorously, such as stem cells, basal skin cells, and gastrointestinal mucosa cells, are also targeted by ionizing radiation. Unrepaired DNA damage often induces apoptosis and cell cycle arrest [14, 15]. Cell apoptosis owing to radiation can be mediated by either p53 or the sphingomyelin/ceramide pathways [16, 17]. In addition, radiation induces mitochondrial fission by activating the MAPK pathway, which promotes the release of apoptotic factors such as cytochrome c [18]. Endothelial cell damage and apoptosis induced by radiation can lead to vascular leak, edema, and the increase in inflammation [14]. Difficulties in repair and regeneration caused by these changes set obstacles to breast reconstruction.

### Indirect cellular damage

Reactive oxygen species (ROS) are an important cause of indirect damage induced by radiation. Ionizing radiation causes the transfer of electrons when it penetrates cells, leading to generation of unstable ROS [19]. In addition to indirectly damaging DNA in the nucleus, ROS also react with key proteins and lipids involved in cell metabolism [20, 21].

Extensive research has found that radiation-induced damage arises at sites outside the irradiated area [22]. This indicates that direct damage of cellular genetic material by penetration of radiation into the nucleus is not a prerequisite for cell damage. Cells damaged by radiation affect bystander cells and cause damage, called the radiation-induced bystander effect, through oxidative metabolism, gap junctions between cells, and paracrine substances [23]. Recent studies have reported that the level of gap junctions is lower between cancer cells than between normal cells, which may mean that it is difficult to specifically kill cancer cells using the bystander effect of radiotherapy [24]. The expansion of radiation damage and poor prognosis caused by the bystander effect are still worthy of attention.

In addition, the radiation-induced inflammatory response is also considered to be important for indirect injury. The acute inflammatory response can cause secondary tissue damage, and the chronic inflammatory response is closely related to radiation-induced soft tissue fibrosis [25].

## Types of soft tissue damage caused by radiotherapy and the underlying mechanisms

In the short term, cell damage caused by ionizing radiation induces an acute inflammatory response, and the associated inflammatory cells are mainly neutrophils and macrophages [26]. Adipocytes that perform certain endocrine functions also exhibit transcriptional regulation of hypoxia-inducible factor (HIF)-1α and secrete a large amount of inflammatory mediators such as vascular endothelial growth factor (VEGF) and interleukin (IL)-6 [27]. The type of acute reaction that appears during the early stage of irradiation depends on the dose and duration of irradiation and is mainly manifested as inflammatory erythema, desquamation, and edema caused by vascular and lymphatic lesions [7].

According to Koenig et al. [28], in addition to acute skin reactions such as transient erythema during the early stage of irradiation, permanent epilation and desquamation can occur approximately 3 weeks after 7 Gy irradiation. Skin atrophy can occur within 12 weeks to 1 year after 10 Gy irradiation, and telangiectasia and pathological fibrosis such as induration may occur after more than 1 year. In severe cases, skin necrosis may occur in the late stage.

Ultrastructural analysis showed that the basal membrane of capillary vessels duplicates and their lumens are ectatic after radiotherapy. Endothelial cells are rich in cytoplasm, including macropinocytotic vesicles and a large number of Weibel-Palade bodies. Many adipocytes are necrotic. A large amount of accumulated collagen and debris from necrotic adipocytes in connective tissue is visible [29].

### Acute inflammation and endothelial injury

**Fig. 1:**
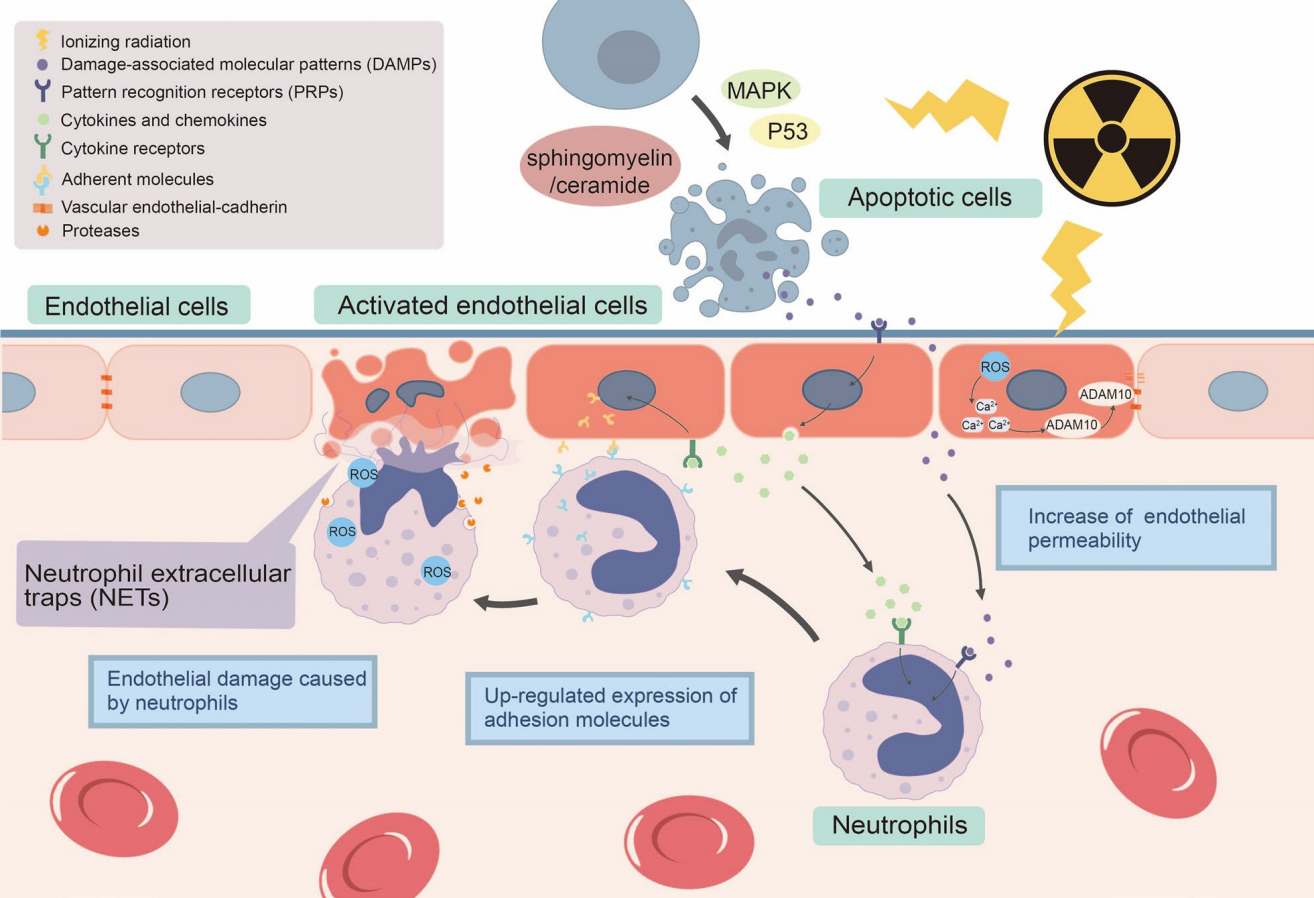
Mechanisms of radiation-induced endothelial damage

The acute phase response is closely related to endothelial cell damage caused by ionizing radiation (Fig. 1). Ionizing radiation-induced apoptosis can release damage-associated molecular patterns (DAMPs), which can act on various pattern recognition receptors (PRPs), not only to activate endothelial cells to switch to a pro-inflammatory phenotype, but also to recruit a series of immune cells to participate in the inflammatory response [30, 31]. Activated endothelial cells secrete cytokines to recruit immune cells and up-regulate the expression levels of adhesion molecules simultaneously to promote the interaction between endothelial cells and immune cells, which results in inflammatory damage to endothelial cells [32]. Protein kinase C (PKC) family is expressed in various cells and is thought to be an important role of neutrophilic and endothelial pro-inflammatory signaling [33, 34]. In neutrophils, PKCδ can activate the NF-κB pathway, which is involved in the secretion of cytokines and chemokines and the production of ROS [35, 36]. In endothelial cells, PKCδ can activate the NF-κB pathway, which is involved in up-regulating the expression of adhesion molecules and promoting the release of inflammatory mediators [34, 37].

Neutrophils play an important role in endothelial injury during acute inflammatory response. The first step in causing damage is to enhance the interaction between neutrophils and endothelial cells. Normal neutrophils have low expression of α4-integrin. Neutrophils under the action of multiple inflammatory factors up-regulate the expression of α4-integrin and enhance the interaction with endothelial cells by acting on VCAM-1 on the surface of endothelial cells. In addition, cytokines such as TNF-α and IL-1 also up-regulate the expression of adhesion molecules such as vascular cell adhesion molecular 1(VCAM-1) and intercellular adhesion molecular 1 (ICAM-1) [37–39]. After completing the adhesion process, neutrophils exert their immune functions and simultaneously damage endothelial cells by secreting proteases, generating reactive oxygen species and forming Neutrophil extracellular traps (NETs) [40–43].

Endothelial cells stimulated by ionizing radiation and inflammatory signals also increase permeability. ROS produced by ionizing radiation raise the concentrations of intracellular Ca^2+^. The increase in intracellular Ca^2+^ activates a disintegrin and metalloprotease 10 (ADAM10), finally causing the degradation of vascular endothelial-cadherin (VE-cadherin) and increasing the endothelial permeability [44–47]. It can be used to explain edema in the acute phase and as one of the pathological basis of fibrosis in the chronic phase.

### Lymphedema

As the most commonly reported complication of breast carcinoma management, the incidence and severity of lymphedema are closely related to axillary radiotherapy and axillary lymph node dissection. However, for most invasive breast cancers, axillary treatment remains indispensable for safety reasons [48]. Three types of lymphedema have been described in breast carcinoma patients, namely arm, truncal, and, in patients who receive breast-conserving therapy, breast lymphedema [49].

There is much speculation and research about the mechanism underlying radiation-induced lymphedema. However, contrary to initial assumptions, early studies performed in vitro and in vivo in humans and other mammals demonstrated that lymphatic vessels are relatively insensitive to radiation [50]. By contrast, lymph nodes are sensitive to radiation [51]. Therefore, lymphedema during breast cancer treatment is attributable to surgical severing of lymphatic vessels and structural changes around lymphatic vessels caused by radiation fibrosis [52]. Ogino et al. [53] elucidated the pathological process of radiotherapy-induced lymphedema. Radiotherapy transforms type I collagen from a random to parallel arrangement and decreases the levels of extracellular matrix (ECM) and type III collagen. The resulting denser spatial structure prevents lymphatic vessels expanding properly and thus hinders lymphatic return and mediates the occurrence of lymphedema.

### Pathological fibrosis and contracture

**Fig. 2:**
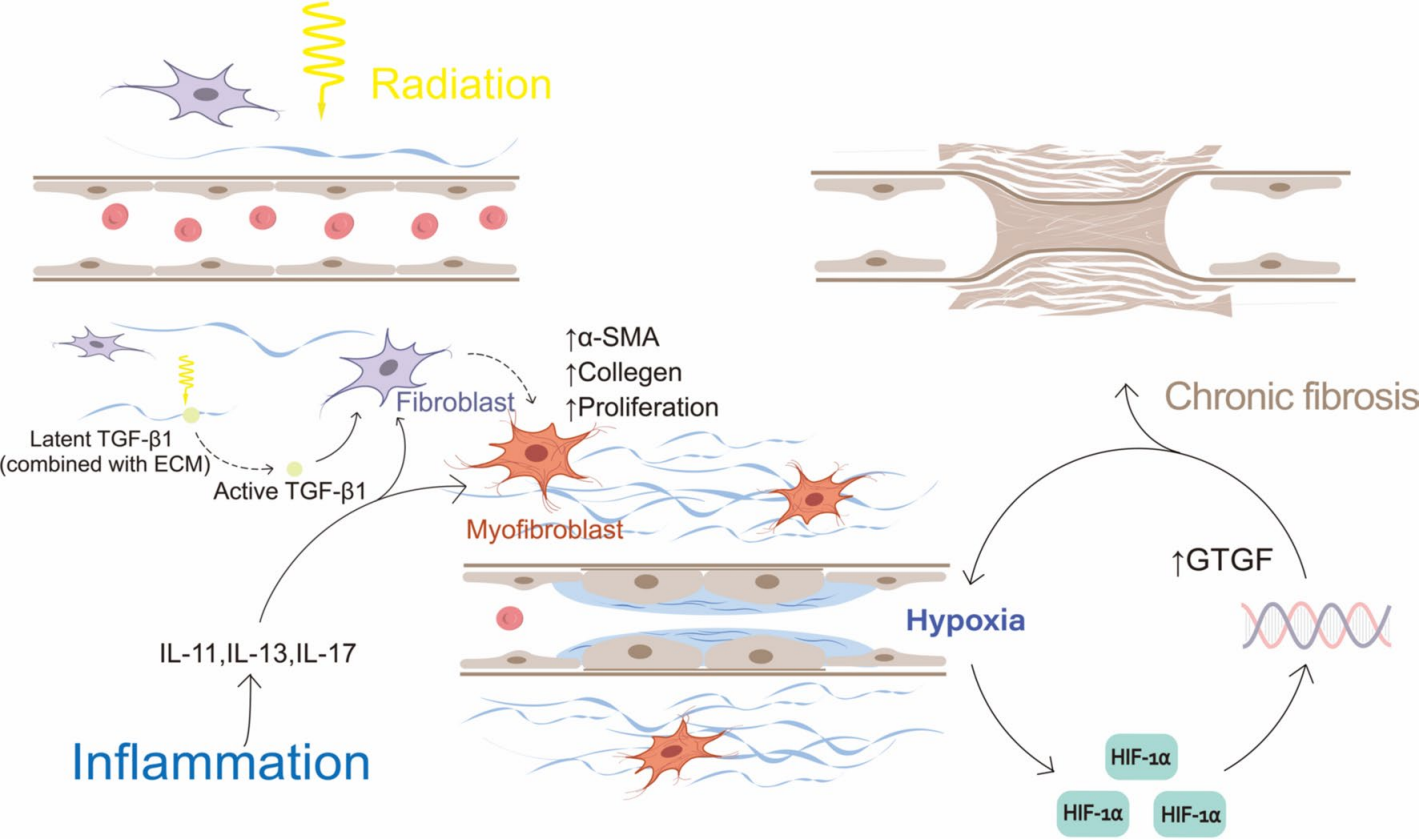
Pathological process of radiofibrosis

Radiation-induced fibrosis results in progressive functional and cosmetic impairment, which is a common late complication of PMRT (Fig. 2). Radiation-induced oxidative lysis releases transforming growth factor β1 (TGFβ1) originally bound to the ECM in a latent form [54]. Owing to the synergistic effect of TGFβ1 and other inflammatory factors such as IL-11, IL-13, and IL-17, fibroblasts that were originally in a static state are transformed into myofibroblasts that exuberantly secrete ECM [26, 55].

Due to the body's repair mechanism, vascular endothelial cells damaged by radiation exhibit high levels of intracellular trafficking and transport between themselves and their environment and high permeability, and vascular basement membrane replication and lumen expansion are also observed. Therefore, the acute phase of injury is mainly manifested as an inflammatory reaction of blood vessels, such as erythema and edema [26]. This leads to deposition of ECM around blood vessels and stenosis of lumens, resulting in ischemia and hypoxia of local tissues. Simultaneously, the shrinkage of the vessel bed caused by radiation damage exacerbates ischemia and hypoxia, which intensifies tissue damage, leading to a vicious circle [29, 56]. Hypoxia caused by radiation induces accumulation of HIF-1α, which is degraded by the ubiquitin-proteasome pathway under normoxic conditions. The transcription factor HIF-1α is the most important regulator of cellular responses to hypoxia and regulates the transcriptional activities of many genes that play important roles in regulating glucose metabolism and promoting angiogenesis, cell migration, ECM deposition, and fibrosis. Fibrosis-related proteins that are transcriptionally regulated by HIF-1α include lipoxidase and connective tissue growth factor [57–60]. In the short term, these changes are conducive to tissue repair, but long-term expression of HIF-1α may cause excessive tissue repair and fibrosis. Relying on the body's natural repair mechanism without treatment can lead to severe fibrosis or tissue necrosis.

In addition, radiotherapy can cause an abnormal morphology of fibroblasts, such as a flattened and enlarged morphology, as well as changes in their biological behavior and synthetic function, leading to modulation of important signaling pathways and adaptive dysfunctions. Fibroblasts injured by radiotherapy exhibit inhibited proliferation but increased migration, invasiveness, adhesion, and contractility. At the same time, activation of the HIF-1α pathway also initiates a local fibrotic response [22]. A study by Rinkevich et al. [61] indicated that fibrosis, during wound healing or radiation fibrosis, in cutaneous tissue is caused by the unique and local lineage of resident fibroblasts, without the contribution of fibroblasts from circulating or other lineages.

Decreased skin compliance due to radiation fibrosis may hinder breast reconstruction, for example, may reduce the retention of grafted fat and increase the probability of capsular contracture in breast implant reconstruction [4]. Elevated skin tension also exacerbates scarring, which greatly affects reconstruction and breast esthetics [62].

## The therapeutic value of adipose-derived stem cells (ADSCs) for breast reconstruction after radiotherapy

**Fig. 3:**
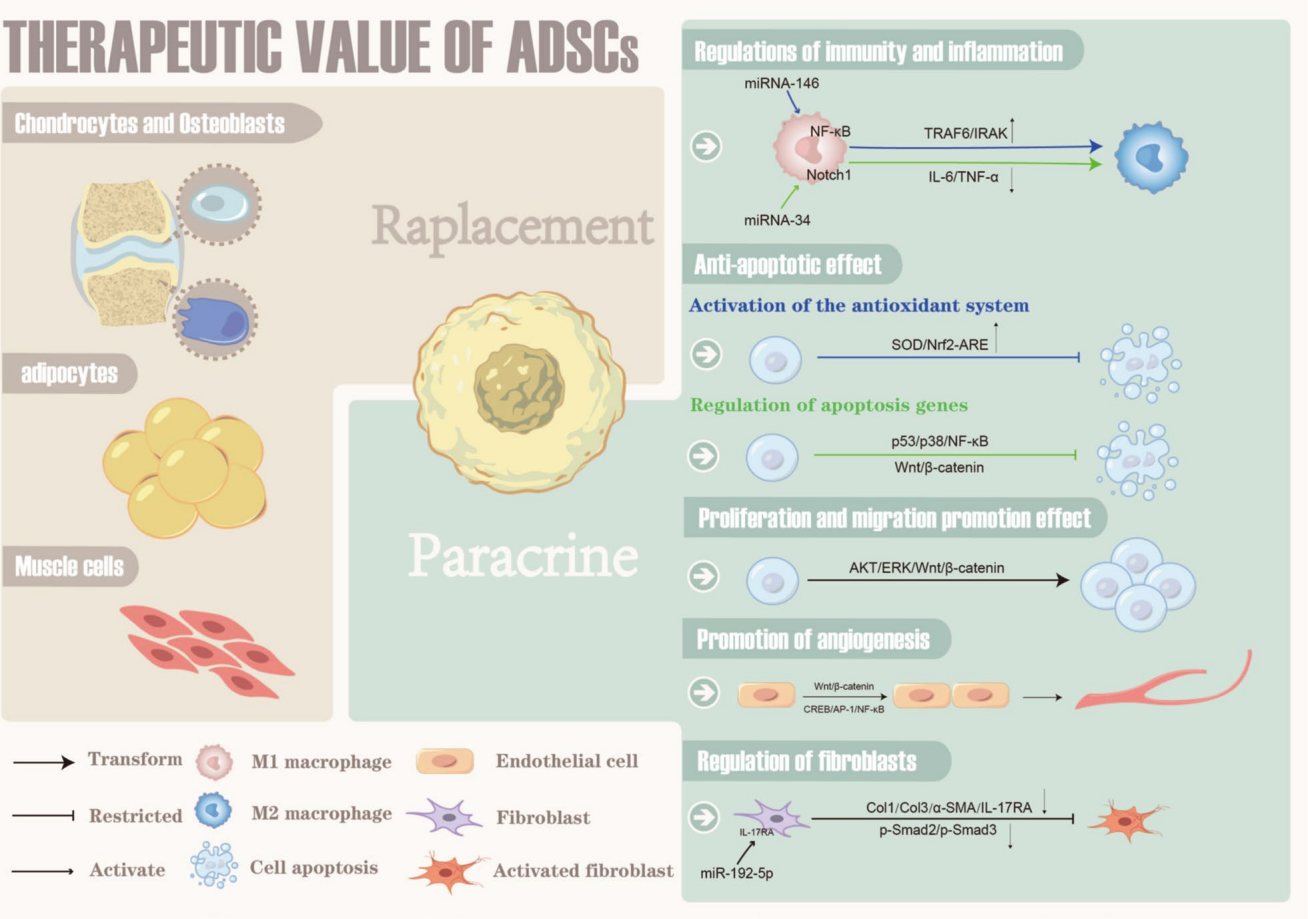
The therapeutic value of ADSCs

In the process of repairing and rebuilding, it is necessary to solve the problem of lack of breast volume and the problem of cell dysfunction and tissue damage caused by radiotherapy. Autologous fat grafting has attracted much attention for these two advantages. It can obviously improve skin tissue suffered from irradiation by improving the compliance and thickness of skin and tissue adhesion [9–11, 54, 63]. A systematic review and meta-analysis published in 2020 confirmed the efficacy and safety of autologous fat transfer for filling defects and improving fibrosis and scar-related conditions [64]. ADSCs are believed to be the most important and potent fat component. Compared with other cell types, such as bone marrow-derived stem cells, ADSCs are favored in the fields of wound healing and tissue regeneration because they are abundant in fat tissue and easily accessible. Additionally, ADSCs appear to be less immunogenic and more genetically stable in long-term culture compared with BMSC [65]. In a clinical study, Rigotti et al. [29] purified Coleman fat to obtain stromal vascular fraction (SVF) and transplanted it into patients who had received radiotherapy. The results indicate that SVF of adipose tissue can induce construction of a new microcirculation. This phenomenon is probably due to the multi-differentiation potential and paracrine effect of ADSCs [66] (Fig. 3).

### Proliferation and differentiation of ADSCs

ADSCs, a type of mesenchymal stem cell (MSC), have the potential of multi-directional differentiation and can differentiate into adipocytes, chondrocytes, osteocytes and endothelial cells. Under specific conditions, ADSCs can replace damaged cells through proliferation and differentiation to repair tissue damage [67, 68].

The mechanisms regulating the proliferation and differentiation of ADSCs are not fully understood. Lin et al. [69] indicated that hypoxia can promote ADSCs differentiation into vascular smooth muscle cells (VMSCs) by mediating Mettl3 gene expression. Isabele et al. [70] elucidated that Col V can enhance the proliferation and differentiation of rabbit ADSCs. Besides, after receiving the stimulation of TGF-β3 and BMP-6, the ADSCs increase the expression of the cartilage formation gene. The study from Denver et al. [71] manifests that the endogenous Notch signal may have the potential to regulate the proliferation, differentiation and bone potential of ADSCs.

### The paracrine effect of ADSCs

ADSCs can secrete various cytokines, chemokines, growth factors, and paracrine molecules in extracellular vesicles and promote cell survival, modulate the inflammatory reaction, and thus enhance regeneration of injured tissue [19, 72].

Many studies have explored the role of MSCs, represented by ADSCs, in repair of radiation injury and other soft tissue injuries (Table 1).

**Table 1:** HMEC: Human microvascular endothelial cell, HUVEC: Human umbilical vein endothelial cell, HaCaT cell: Human keratinocyte, HDLEC: Human dermal lymphatic endothelial cell, LEC: Lymphangial endothelial cell, hucMSC-Evs: Extracellular vesicles of human umbilical cord mesenchymal stem cells; ADSC-MVs: Microvesicles of ADSCs, PRP: Platelet-rich plasma, HKFs: Human keloid fibroblasts, ADSCC-CM: Adipose-derived stem cell concentrated conditioned medium, cGVHD: Chronic graft-versus-host disease, PDGF-ADSC-EVs: Extracellular vesicles of PDGF-treated ADSCs, MSC-Exos: Exosomes of mesenchymal stem cells, BM-MSC: Bone marrow-derived mesenchymal stem cell, HucMSC-Exos: Exosomes of human umbilical cord mesenchymal stem cells, ADSC-Exos: Exosomes of ADSCs, MSC-EVs, Extracellular vesicles of mesenchymal stem cells, α-SMA: α-smooth muscle actin, ESCs: Endometrial epithelial cells

Model	In vivo/in vitro	Method	Therapeutic effect	Reference
Irradiation model in rats	In vivo	BM-MSC-derived exosomes	**Regulates differentiation** and survival of other mesenchymal stem cells **Anti-oxidative stress** **Anti-apoptotic**	Zuo et al. [73]
SCID mice	In vivo	Transplantation of HMECs treated with PDGF-ADSC-EVs	**Angiogenesis** of endothelial cells	Tatiana et al. [74]
HUVECs, HaCaT cells, fibroblasts, and wound healing model in BALB/c mice	Both	In vitro: co-culture with ADSC-MVs In vivo: subcutaneous injection of ADSC-MVs	**Promotes proliferation:** promotes re-epithelialization, collagen deposition, and angiogenesis at the wound site	Ren et al. [77]
Model of skin lesions under oxidative stress using HaCaT cells	In vitro	Co-culture of HaCaT cells with ADSC-Exos	**Promotes proliferation and migration** **Anti-apoptotic**	Ma et al. [78]
Skin lesion model exposing to hydrogen peroxide (H_2_O_2_)	In vitro	Co-culture of HaCaT cells with ADSC-Exos	**Promotes proliferation and migration** **Anti-apoptotic**	He et al. [79]
Skin burn model in rats	In vitro	Co-culture of HUVECs with HucMSC-Exos	**Angiogenesis** of endothelial cells	Zhang et al. [80]
cGVHD mouse model	In vivo	Intraperitoneal injection of hucMSC-Evs	**Regulates immunity:** suppresses activation of the immune response in macrophages and B cells	Guo et al. [82]
HKFs and hypertrophic scar model in rabbit ear	Both	Transplantation of lyophilized ADSCC-CM combined with a polysaccharide hydrogel	**Regulates associated cellular behavior:** downregulates α-SMA expression in HKFs in a dose-dependent manner to prevent hypertrophic scarring	Zhang et al. [83]
Ultraviolet irradiation model in mice	Both	In vitro: co-culture with MSC-Exos In vivo: injection of MSC-Exos	**Anti-oxidative stress**	Wang et al. [93]
SJL mice	Both	In vitro: co-culture with MSC-Ex In vivo: intravenous injection of MSC-EVs	**Anti-apoptotic.** Stimulates bone marrow hematopoietic cells to restore hematopoiesis and reverse radiation-induced apoptosis	Wen et al. [94]
HDLECs	In vitro	Co-culture with ADSCs	**Promote proliferation and migration of LECs**	Saijo et al. [100]
Secondary lymphedema model in C57BL/6J mice	In vivo	Transplantation of ADSCs	**Promotes proliferation of LECs** and improves fibrosis and the expansion capacity of lymphatic vessels	Ogino et al. [53]
Radiation-induced vaginal injury in rats	In vivo	Implantation of a protein scaffold loaded with ADSCs into injury sites	**Promotes proliferation:** promotes regeneration of vaginal epithelial cells and repairs and improves vaginal stenosis and contractures of vaginal tissue	Ye et al. [101]
Wound healing model in C57BL/6 mice	In vivo	Transplantation of PRP combined with ADSCs	PRP can promote migration of ADSCs through the Rho GTP-LIMK1-Cofilin signaling pathway	Zhang et al. [102]

ADSCs can increase proliferation and survival of specific stem or progenitor cells in tissues and organs [73] and promote angiogenesis and lymphangiogenesis in damaged tissue [74–76]. The mechanism underlying ADSC-mediated promotion of cell proliferation is unclear, but studies suggest that activation of the AKT, ERK, and Wnt/β-catenin signaling pathways is closely related to this effect [73, 77–79]. Wnt4 carried in exosomes released by ADSCs induces β-catenin activation and elicits a proangiogenic effect in endothelial cells [80]. In addition, vesicles released by ADSCs can directly carry nuclear factor kappa-B (NF-κB) and thereby activate the NF-κB signaling pathway in endothelial cells and promote angiogenesis [81]. ADSCs can also regulate the biological behavior of immune cells and fibroblasts and thereby prevent radiation fibrosis to a certain extent [82–84]. In the TGF-β1-induced endometrial fibrosis damage model, ADSC-Exosomes can regulate the miRNA-150-5p by raising the expression level of lncRNA-MIAT, which is related to fibrosis [85]. Several studies have shown that some microRNAs (miRNAs) and cytokines carried by ADSC-derived extracellular vesicles can alter the secretion profile of macrophages and transform their phenotype [86–90]. For example, miRNA-146 and miRNA-34 can induce macrophages to switch from a M1- to M2-like phenotype. MiRNA146 [91] upregulates M2-related genes such as *TRAF6* and *IRAK* through the NF-κB pathway, while miRNA-34 [92] inhibits expression of the M1-related genes *IL-6* and *TNF-α* by targeting the Notch1 pathway. The anti-oxidative stress [93] and anti-apoptotic [94] effects of substances secreted by ADSCs are critical for repairing radiation-induced DNA damage in the acute phase and preventing the exacerbation of damage caused by the bystander effect. ADSC can effectively activate the antioxidant system, such as superoxide dismutase (SOD) [95, 96], Nrf2-antioxidant response element (ARE) pathway [97–99] and so on.

## Repairing radiation damage with ADSCs: perspective and challenges

Studies suggest that ADSCs exert their biological effects mainly through cargoes carried in extracellular vesicles (exosomes or microparticles) and soluble secreted factors [19]. There are many types of cargoes, and it is insufficient to simply clarify the mechanism underlying the effects of stem cell therapy on radiation damage and the intertwined signaling network [103]. Generation of extracellular vesicles with a single phenotype using specific environmental stimuli or interventional methods and performing in vivo treatment with these vesicles can improve the efficacy and safety of stem cell therapy. Stem cells exposed to radiation highly express the CD29/CD81 complex, making it easier to isolate extracellular vesicles [104]. Treatment of ischemia-related injury can be improved using microvesicles derived from stem cells exposed to hypoxia [74]. Although ADSCs is easy to obtain from adipose tissues and is easy to cultivate in vitro, there are still some studies indicate that ADSCs transplanting and surviving in vivo need more complicated conditions. The survival of ADSCs after injection is not satisfying [105–107]. The co-transplantation between ADSCs and adaptive biomaterial scaffolds can provide an ideal environment for cell survival and promote the adhesion, proliferation and differentiation of ADSCs. Use of a combination of ADSCs and PRP promotes wound healing, granulation, collagen deposition, and re-epithelialization [102]. Mixed implantation of cell scaffolds with good biocompatibility and ADSCs can prolong the duration of cytokine secretion by these cells [101]. A sheet of ADSCs can achieve better effects than injection of a suspension of ADSCs for treatment of chronic ulcer wounds. Prevention of pericyte escape to a certain extent by a sheet of ADSCs stabilizes angiogenesis, promotes granulation tissue reabsorption, and inhibits scarring. In addition, a sheet of ADSCs more obviously promotes regeneration of skin accessory structures such as hair follicles [108]. In addition, the recruitment of endogenous ADSCs is also a valuable research direction. Li et al. [109] applicated the external force to regulate the tissue stiffness, which can affect the migration and differentiation of ADSCS.

Finally, most current research about ADSCs and other MSCs mainly focuses on curative effects in the acute phase of radiation injury, and there is little research about treatment of chronic phase complications. For breast reconstruction, most patients are in the chronic phase of radiation damage, and fibrosis is relatively severe. The efficacy of stem cell therapy to improve skin fibrosis and skin compliance and the underlying mechanism require further research.

## Conclusion

ADSCs can promote wound healing and tissue repair. However, the mechanism by which ADSCs improve radiation injury and their ideal application method in this context must be studied. Further research is also needed to determine whether ADSC therapy is efficacious and safe for breast reconstruction after radiotherapy and, if so, to examine the underlying mechanism.

## Data Availability

Not applicable.

## Abbreviations

PMRT: Postmastectomy radiotherapy
ROS: Reactive oxygen species
HIF-1α: Hypoxia-inducible factor-1α
VEGF: Vascular endothelial growth factor
IL-6: Interleukin-6
DAMPs: Damage-associated molecular patterns
PRPs: Pattern recognition receptors
PKC: Protein kinase C
VCAM-1: Vascular cell adhesion molecular 1
ICAM-1: Intercellular adhesion molecular 1
NETs: Neutrophil extracellular traps
ADAM10: A disintegrin and metalloprotease 10
VE-cadherin: Vascular endothelial-cadherin
miRNA: Micro RNA
lncRNA: Long noncoding RNA
SOD: Superoxide dismutase
Nrf2: Nuclear factor erythroid 2-related factor-2
ARE: Antioxidant response element
ECM: Extracellular matrix
TGFβ1: Transforming growth factor β1
SVF: Stromal vascular fraction
ADSCs: Adipose-derived stem cells
VMSCs: Vascular smooth muscle cells
MSC: Mesenchymal stem cell
NF-κB: Nuclear factor kappa-B
HMEC: Human microvascular endothelial cell
HUVEC: Human umbilical vein endothelial cell
HaCaT cell: Human keratinocyte
HDLEC: Human dermal lymphatic endothelial cell
LEC: Lymphangial endothelial cell
hucMSC-Evs: Extracellular vesicles of human umbilical cord mesenchymal stem cells
ADSC-MVs: Microvesicles of ADSC
PRP: Platelet-rich plasma
HKFs: Human keloid fibroblasts
ADSCC-CM: Adipose-derived stem cell concentrated conditioned medium
cGVHD: Chronic graft-versus-host disease
PDGF-ADSC-EVs: Extracellular vesicles of PDGF-treated ADSCs
MSC-Ex: Exosomes of mesenchymal stem cells
BM-MSC: Bone marrow-derived mesenchymal stem cell
HucMSC-Ex: Exosomes of human umbilical cord mesenchymal stem cells
ADSC-ex: Exosomes of ADSCs
MSC-EVs: Extracellular vesicles of mesenchymal stem cells
α-SMA: α-Smooth muscle actin

## References

[1] Early Breast Cancer Trialists' Collaborative Group (2000). Favourable and unfavourable effects on long-term survival of radiotherapy for early breast cancer: an overview of the randomised trials. Lancet..

[2] Jugenburg M, Disa JJ, Pusic AL, Cordeiro PG (2007). Impact of radiotherapy on breast reconstruction. Clin Plast Surg..

[3] Yun JH, Diaz R, Orman AG (2018). Breast reconstruction and radiation therapy. Cancer Control..

[4] Gerber B, Marx M, Untch M, Faridi A (2015). Breast reconstruction following cancer treatment. Dtsch Arztebl Int..

[5] Krueger EA, Wilkins EG, Strawderman M, Cederna P, Goldfarb S, Vicini FA (2001). Complications and patient satisfaction following expander/implant breast reconstruction with and without radiotherapy. Int J Radiat Oncol Biol Phys.

[6] Nava MB, Pennati AE, Lozza L, Spano A, Zambetti M, Catanuto G (2011). Outcome of different timings of radiotherapy in implant-based breast reconstructions. Plast Reconstr Surg..

[7] Kronowitz SJ, Robb GL (2004). Breast reconstruction with postmastectomy radiation therapy: current issues. Plast Reconstr Surg..

[8] Adesiyun TA, Lee BT, Yueh JH, Chen C, Colakoglu S, Anderson KEM (2011). Impact of sequencing of postmastectomy radiotherapy and breast reconstruction on timing and rate of complications and patient satisfaction. Int J Radiat Oncol* Biol* Phys..

[9] Borrelli MR, Shen AH, Lee GK, Momeni A, Longaker MT, Wan DC (2019). Radiation-induced skin fibrosis: pathogenesis, current treatment options, and emerging therapeutics. Ann Plast Surg..

[10] Fukuba M, Uozaki H, Komuro Y (2020). Effectiveness of the combination of fat grafting and injection on radiation ulcer healing. J Plast Surg Hand Surg..

[11] Garza RM, Paik KJ, Chung MT, Duscher D, Gurtner GC, Longaker MT (2014). Studies in fat grafting: Part III. Fat grafting irradiated tissue—improved skin quality and decreased fat graft retention. Plast Reconstr Surg..

[12] Nepon H, Safran T, Reece EM, Murphy AM, Vorstenbosch J, Davison PG (2021). Radiation-induced tissue damage: clinical consequences and current treatment options. Semin Plast Surg..

[13] Brown KR, Rzucidlo E (2011). Acute and chronic radiation injury. J Vasc Surg..

[14] Panganiban RA, Mungunsukh O, Day RM (2013). X-irradiation induces ER stress, apoptosis, and senescence in pulmonary artery endothelial cells. Int J Radiat Biol..

[15] Wang H, Dong J, Li G, Tan Y, Zhao H, Zhang L (2021). The small protein MafG plays a critical role in MC3T3-E1 cell apoptosis induced by simulated microgravity and radiation. Biochem Biophys Res Commun..

[16] Venkatesulu BP, Mahadevan LS, Aliru ML, Yang X, Bodd MH, Singh PK (2018). Radiation-induced endothelial vascular injury: a review of possible mechanisms. JACC Basic Transl Sci..

[17] Seideman JH, Stancevic B, Rotolo JA, McDevitt MR, Howell RW, Kolesnick RN (2011). Alpha particles induce apoptosis through the sphingomyelin pathway. Radiat Res..

[18] Jin X, Li F, Liu B, Zheng X, Li H, Ye F (2018). Different mitochondrial fragmentation after irradiation with X-rays and carbon ions in HeLa cells and its influence on cellular apoptosis. Biochem Biophys Res Commun..

[19] Wang KX, Cui WW, Yang X, Tao AB, Lan T, Li TS (2021). Mesenchymal stem cells for mitigating radiotherapy side effects. Cells-Basel..

[20] Hubenak JR, Zhang Q, Branch CD, Kronowitz SJ (2014). Mechanisms of injury to normal tissue after radiotherapy: a review. Plast Reconstr Surg..

[21] Kammeyer A, Luiten RM (2015). Oxidation events and skin aging. Ageing Res Rev..

[22] Shukla L, Luwor R, Ritchie ME, Akbarzadeh S, Zhu HJ, Morrison W (2020). Therapeutic reversal of radiotherapy injury to pro-fibrotic dysfunctional fibroblasts in vitro using adipose-derived stem cells. Plast Reconstr Surg Glob Open..

[23] Azzam EI, de Toledo SM, Little JB (2003). Oxidative metabolism, gap junctions and the ionizing radiation-induced bystander effect. Oncogene..

[24] Wan C, Sun Y, Tian Y, Lu L, Dai X, Meng J (2020). Irradiated tumor cell-derived microparticles mediate tumor eradication via cell killing and immune reprogramming. Sci Adv..

[25] De Ruysscher D, Niedermann G, Burnet NG, Siva S, Lee A, Hegi-Johnson F (2019). Radiotherapy toxicity. Nat Rev Dis Primers..

[26] Ejaz A, Greenberger JS, Rubin PJ (2019). Understanding the mechanism of radiation induced fibrosis and therapy options. Pharmacol Ther..

[27] Sun K, Wernstedt AI, Kusminski CM, Bueno AC, Wang ZV, Pollard JW (2012). Dichotomous effects of VEGF-A on adipose tissue dysfunction. Proc Natl Acad Sci USA.

[28] Koenig TR, Wolff D, Mettler FA, Wagner LK (2001). Skin injuries from fluoroscopically guided procedures: part 1, characteristics of radiation injury. AJR Am J Roentgenol..

[29] Rigotti G, Marchi A, Galie M, Baroni G, Benati D, Krampera M (2007). Clinical treatment of radiotherapy tissue damage by lipoaspirate transplant: a healing process mediated by adipose-derived adult stem cells. Plast Reconstr Surg..

[30] Gong T, Liu L, Jiang W, Zhou R (2020). DAMP-sensing receptors in sterile inflammation and inflammatory diseases. Nat Rev Immunol..

[31] Denning NL, Aziz M, Gurien SD, Wang P (2019). DAMPs and NETs in sepsis. Front Immunol..

[32] Wijerathne H, Langston JC, Yang Q, Sun S, Miyamoto C, Kilpatrick LE (2021). Mechanisms of radiation-induced endothelium damage: emerging models and technologies. Radiother Oncol..

[33] Soroush F, Tang Y, Guglielmo K, Engelmann A, Liverani E, Patel A (2019). Protein kinase C-delta (PKCdelta) tyrosine phosphorylation is a critical regulator of neutrophil-endothelial cell interaction in inflammation. Shock..

[34] Soroush F, Zhang T, King DJ, Tang Y, Deosarkar S, Prabhakarpandian B (2016). A novel microfluidic assay reveals a key role for protein kinase C delta in regulating human neutrophil-endothelium interaction. J Leukoc Biol..

[35] Kilpatrick LE, Sun S, Li H, Vary TC, Korchak HM (2010). Regulation of TNF-induced oxygen radical production in human neutrophils: role of delta-PKC. J Leukoc Biol..

[36] Drost EM, MacNee W (2002). Potential role of IL-8, platelet-activating factor and TNF-alpha in the sequestration of neutrophils in the lung: effects on neutrophil deformability, adhesion receptor expression, and chemotaxis. Eur J Immunol..

[37] Zhong L, Simard MJ, Huot J (2018). Endothelial microRNAs regulating the NF-kappaB pathway and cell adhesion molecules during inflammation. Faseb J..

[38] Ibbotson GC, Doig C, Kaur J, Gill V, Ostrovsky L, Fairhead T (2001). Functional alpha4-integrin: a newly identified pathway of neutrophil recruitment in critically ill septic patients. Nat Med..

[39] Schunk SJ, Triem S, Schmit D, Zewinger S, Sarakpi T, Becker E (2021). Interleukin-1alpha Is a central regulator of leukocyte-endothelial adhesion in myocardial infarction and in chronic kidney disease. Circulation..

[40] Brinkmann V, Reichard U, Goosmann C, Fauler B, Uhlemann Y, Weiss DS (2004). Neutrophil extracellular traps kill bacteria. Science..

[41] Kang L, Yu H, Yang X, Zhu Y, Bai X, Wang R (2020). Neutrophil extracellular traps released by neutrophils impair revascularization and vascular remodeling after stroke. Nat Commun..

[42] Thiam HR, Wong SL, Wagner DD, Waterman CM (2020). Cellular mechanisms of NETosis. Annu Rev Cell Dev Biol..

[43] Xu J, Zhang X, Pelayo R, Monestier M, Ammollo CT, Semeraro F (2009). Extracellular histones are major mediators of death in sepsis. Nat Med..

[44] Kouam PN, Rezniczek GA, Adamietz IA, Buhler H (2019). Ionizing radiation increases the endothelial permeability and the transendothelial migration of tumor cells through ADAM10-activation and subsequent degradation of VE-cadherin. Bmc Cancer..

[45] Schulz B, Pruessmeyer J, Maretzky T, Ludwig A, Blobel CP, Saftig P (2008). ADAM10 regulates endothelial permeability and T-Cell transmigration by proteolysis of vascular endothelial cadherin. Circ Res..

[46] Flemming S, Burkard N, Renschler M, Vielmuth F, Meir M, Schick MA (2015). Soluble VE-cadherin is involved in endothelial barrier breakdown in systemic inflammation and sepsis. Cardiovasc Res..

[47] Gavard J (2014). Endothelial permeability and VE-cadherin: a wacky comradeship. Cell Adhes Migr..

[48] Rockson SG (2018). Lymphedema after breast cancer treatment. N Engl J Med..

[49] Meek AG (1998). Breast radiotherapy and lymphedema. Cancer-Am Cancer Soc..

[50] Lenzi M, Bassani G (1963). The effect of radiation on the lymph and on the lymph vessels. Radiology..

[51] Fajardo LF (1994). Effects of ionizing radiation on lymph nodes. A review. Front Radiat Ther Oncol..

[52] Tashiro K, Feng J, Wu SH, Mashiko T, Kanayama K, Narushima M (2017). Pathological changes of adipose tissue in secondary lymphoedema. Br J Dermatol..

[53] Ogino R, Hayashida K, Yamakawa S, Morita E (2020). Adipose-derived stem cells promote intussusceptive lymphangiogenesis by restricting dermal fibrosis in irradiated tissue of mice. Int J Mol Sci..

[54] Kumar R, Griffin M, Adigbli G, Kalavrezos N, Butler PE (2016). Lipotransfer for radiation-induced skin fibrosis. Br J Surg..

[55] Henderson NC, Rieder F, Wynn TA (2020). Fibrosis: from mechanisms to medicines. Nature..

[56] Phulpin B, Gangloff P, Tran N, Bravetti P, Merlin JL, Dolivet G (2009). Rehabilitation of irradiated head and neck tissues by autologous fat transplantation. Plast Reconstr Surg..

[57] Darby IA, Hewitson TD (2016). Hypoxia in tissue repair and fibrosis. Cell Tissue Res..

[58] Wang Q, Wang P, Qin Z, Yang X, Pan B, Nie F (2021). Altered glucose metabolism and cell function in keloid fibroblasts under hypoxia. Redox Biol..

[59] Yeo EJ (2019). Hypoxia and aging. Exp Mol Med..

[60] Halberg N, Khan T, Trujillo ME, Wernstedt-Asterholm I, Attie AD, Sherwani S (2009). Hypoxia-inducible factor 1alpha induces fibrosis and insulin resistance in white adipose tissue. Mol Cell Biol..

[61] Rinkevich Y, Walmsley GG, Hu MS, Maan ZN, Newman AM, Drukker M (2015). Skin fibrosis. Identification and isolation of a dermal lineage with intrinsic fibrogenic potential. Science..

[62] Mascharak S, DesJardins-Park HE, Davitt MF, Griffin M, Borrelli MR, Moore AL (2021). Preventing Engrailed-1 activation in fibroblasts yields wound regeneration without scarring. Science..

[63] Glass GE, Ferretti P (2019). Adipose-derived stem cells in aesthetic surgery. Aesthet Surg J..

[64] Krastev TK, Schop SJ, Hommes J, Piatkowski A, van der Hulst R (2020). Autologous fat transfer to treat fibrosis and scar-related conditions: a systematic review and meta-analysis. J Plast Reconstr Aesthet Surg..

[65] Guo J, Hu H, Gorecka J, Bai H, He H, Assi R (2018). Adipose-derived mesenchymal stem cells accelerate diabetic wound healing in a similar fashion as bone marrow-derived cells. Am J Physiol Cell Physiol..

[66] Shukla L, Yuan Y, Shayan R, Greening DW, Karnezis T (2020). Fat Therapeutics: the clinical capacity of adipose-derived stem cells and exosomes for human disease and tissue regeneration. Front Pharmacol..

[67] Huang Q, Zou Y, Arno MC, Chen S, Wang T, Gao J (2017). Hydrogel scaffolds for differentiation of adipose-derived stem cells. Chem Soc Rev..

[68] Labusca L, Herea DD, Emanuela MA, Stavila C, Danceanu C, Plamadeala P (2021). Magnetic nanoparticles and magnetic field exposure enhances chondrogenesis of human adipose derived mesenchymal stem cells but not of Wharton jelly mesenchymal stem cells. Front Bioeng Biotechnol..

[69] Lin J, Zhu Q, Huang J, Cai R, Kuang Y (2020). Hypoxia promotes vascular smooth muscle cell (VSMC) differentiation of adipose-derived stem cell (ADSC) by regulating Mettl3 and paracrine factors. Stem Cells Int..

[70] Brindo DCI, Velosa A, Carrasco S, Dos SFA, Tomaz DMJ, Pompeu E (2021). Post-adipose-derived stem cells (ADSC) stimulated by collagen type V (Col V) mitigate the progression of osteoarthritic rabbit articular cartilage. Front Cell Dev Biol..

[71] Lough DM, Chambers C, Germann G, Bueno R, Reichensperger J, Swanson E (2016). Regulation of ADSC osteoinductive potential using notch pathway inhibition and gene rescue: a potential on/off switch for clinical applications in bone formation and reconstructive efforts. Plast Reconstr Surg..

[72] Zanoni M, Cortesi M, Zamagni A, Tesei A (2019). The role of mesenchymal stem cells in radiation-induced lung fibrosis. Int J Mol Sci..

[73] Zuo R, Liu M, Wang Y, Li J, Wang W, Wu J (2019). BM-MSC-derived exosomes alleviate radiation-induced bone loss by restoring the function of recipient BM-MSCs and activating Wnt/beta-catenin signaling. Stem Cell Res Ther..

[74] Lopatina T, Bruno S, Tetta C, Kalinina N, Porta M, Camussi G (2014). Platelet-derived growth factor regulates the secretion of extracellular vesicles by adipose mesenchymal stem cells and enhances their angiogenic potential. Cell Commun Signal..

[75] Hu LR, Pan J (2020). Adipose-derived stem cell therapy shows promising results for secondary lymphedema. World J Stem Cells..

[76] Wu SC, Kuo PJ, Rau CS, Huang LH, Lin CW, Wu YC (2021). Increased angiogenesis by exosomes secreted by adipose-derived stem cells upon lipopolysaccharide stimulation. Int J Mol Sci..

[77] Ren S, Chen J, Duscher D, Liu Y, Guo G, Kang Y (2019). Microvesicles from human adipose stem cells promote wound healing by optimizing cellular functions via AKT and ERK signaling pathways. Stem Cell Res Ther..

[78] Ma T, Fu B, Yang X, Xiao Y, Pan M (2019). Adipose mesenchymal stem cell-derived exosomes promote cell proliferation, migration, and inhibit cell apoptosis via Wnt/beta-catenin signaling in cutaneous wound healing. J Cell Biochem..

[79] He L, Zhu C, Jia J, Hao XY, Yu XY, Liu XY (2020). ADSC-Exos containing MALAT1 promotes wound healing by targeting miR-124 through activating Wnt/beta-catenin pathway. Biosci Rep..

[80] Zhang B, Wu X, Zhang X, Sun Y, Yan Y, Shi H (2015). Human umbilical cord mesenchymal stem cell exosomes enhance angiogenesis through the Wnt4/beta-catenin pathway. Stem Cells Transl Med..

[81] Anderson JD, Johansson HJ, Graham CS, Vesterlund M, Pham MT, Bramlett CS (2016). Comprehensive proteomic analysis of mesenchymal stem cell exosomes reveals modulation of angiogenesis via nuclear factor-KappaB signaling. Stem Cells..

[82] Guo L, Lai P, Wang Y, Huang T, Chen X, Geng S (2020). Extracellular vesicles derived from mesenchymal stem cells prevent skin fibrosis in the cGVHD mouse model by suppressing the activation of macrophages and B cells immune response. Int Immunopharmacol..

[83] Zhang C, Wang T, Zhang L, Chen P, Tang S, Chen A (2021). Combination of lyophilized adipose-derived stem cell concentrated conditioned medium and polysaccharide hydrogel in the inhibition of hypertrophic scarring. Stem Cell Res Ther..

[84] Li Y, Zhang J, Shi J, Liu K, Wang X, Jia Y (2021). Exosomes derived from human adipose mesenchymal stem cells attenuate hypertrophic scar fibrosis by miR-192-5p/IL-17RA/Smad axis. Stem Cell Res Ther..

[85] Shao X, Qin J, Wan C, Cheng J, Wang L, Ai G (2021). ADSC exosomes mediate lncRNA-MIAT alleviation of endometrial fibrosis by regulating miR-150-5p. Front Genet..

[86] Kruger MJ, Conradie MM, Conradie M, van de Vyver M (2018). ADSC-conditioned media elicit an ex vivo anti-inflammatory macrophage response. J Mol Endocrinol..

[87] Ucal M, Maurer C, Etschmaier V, Hamberger D, Grunbacher G, Togl L (2021). Inflammatory pre-conditioning of adipose-derived stem cells with cerebrospinal fluid from traumatic brain injury patients alters the immunomodulatory potential of ADSC secretomes. J Neurotrauma..

[88] Zhao H, Shang Q, Pan Z, Bai Y, Li Z, Zhang H (2018). Exosomes from adipose-derived stem cells attenuate adipose inflammation and obesity through polarizing M2 macrophages and beiging in white adipose tissue. Diabetes..

[89] Deng S, Zhou X, Ge Z, Song Y, Wang H, Liu X (2019). Exosomes from adipose-derived mesenchymal stem cells ameliorate cardiac damage after myocardial infarction by activating S1P/SK1/S1PR1 signaling and promoting macrophage M2 polarization. Int J Biochem Cell Biol..

[90] Yang CY, Chang PY, Chen JY, Wu BS, Yang AH, Lee OK (2021). Adipose-derived mesenchymal stem cells attenuate dialysis-induced peritoneal fibrosis by modulating macrophage polarization via interleukin-6. Stem Cell Res Ther..

[91] Vergadi E, Vaporidi K, Theodorakis EE, Doxaki C, Lagoudaki E, Ieronymaki E (2014). Akt2 deficiency protects from acute lung injury via alternative macrophage activation and miR-146a induction in mice. J Immunol..

[92] Jiang P, Liu R, Zheng Y, Liu X, Chang L, Xiong S (2012). MiR-34a inhibits lipopolysaccharide-induced inflammatory response through targeting Notch1 in murine macrophages. Exp Cell Res..

[93] Wang T, Jian Z, Baskys A, Yang J, Li J, Guo H (2020). MSC-derived exosomes protect against oxidative stress-induced skin injury via adaptive regulation of the NRF2 defense system. Biomaterials..

[94] Wen S, Dooner M, Cheng Y, Papa E, Del TM, Pereira M (2016). Mesenchymal stromal cell-derived extracellular vesicles rescue radiation damage to murine marrow hematopoietic cells. Leukemia..

[95] Rajendran NK, Houreld NN, Abrahamse H (2021). In vitro wound healing potential of photobiomodulation is possibly mediated by its stimulatory effect on AKT expression in adipose-derived stem cells. Oxid Med Cell Longev..

[96] Wu SH, Yu JH, Liao YT, Liu KH, Chiang ER, Chang MC (2022). Comparison of the infant and adult adipose-derived mesenchymal stem cells in proliferation, senescence, anti-oxidative ability and differentiation potential. Tissue Eng Regen Med..

[97] Huang T, Tong H, Zhou H, Wang J, Hu L, Wang Y (2022). ADSC-exosomes alleviate MTX-induced rat neuronal damage by activating Nrf2-ARE pathway. J Mol Neurosci..

[98] Li L, Ngo H, Hwang E, Wei X, Liu Y, Liu J (2019). Conditioned medium from human adipose-derived mesenchymal stem cell culture prevents UVB-induced skin aging in human keratinocytes and dermal fibroblasts. Int J Mol Sci..

[99] Chen X, Yan L, Guo Z, Chen Z, Chen Y, Li M (2016). Adipose-derived mesenchymal stem cells promote the survival of fat grafts via crosstalk between the Nrf2 and TLR4 pathways. Cell Death Dis..

[100] Saijo H, Suzuki K, Yoshimoto H, Imamura Y, Yamashita S, Tanaka K (2019). Paracrine effects of adipose-derived stem cells promote lymphangiogenesis in irradiated lymphatic endothelial cells. Plast Reconstr Surg..

[101] Ye M, Yu L, She Y, Wang S, Wang M, Zhao Q (2020). Healing effects of a protein scaffold loaded with adipose-derived mesenchymal stem cells on radiation-induced vaginal injury in rats. J Int Med Res..

[102] Zhang L, Zhang B, Liao B, Yuan S, Liu Y, Liao Z (2019). Platelet-rich plasma in combination with adipose-derived stem cells promotes skin wound healing through activating Rho GTPase-mediated signaling pathway. Am J Transl Res..

[103] Kalluri R, LeBleu VS (2020). The biology, function, and biomedical applications of exosomes. Science..

[104] Hazawa M, Tomiyama K, Saotome-Nakamura A, Obara C, Yasuda T, Gotoh T (2014). Radiation increases the cellular uptake of exosomes through CD29/CD81 complex formation. Biochem Biophys Res Commun..

[105] Louwen F, Ritter A, Kreis NN, Yuan J (2018). Insight into the development of obesity: functional alterations of adipose-derived mesenchymal stem cells. Obes Rev..

[106] Hutton DL, Grayson WL (2016). Hypoxia inhibits de novo vascular assembly of adipose-derived stromal/stem cell populations, but promotes growth of preformed vessels. Tissue Eng Part A..

[107] Dong Z, Peng Z, Chang Q, Lu F (2013). The survival condition and immunoregulatory function of adipose stromal vascular fraction (SVF) in the early stage of nonvascularized adipose transplantation. PLoS ONE..

[108] Alexandrushkina N, Nimiritsky P, Eremichev R, Popov V, Arbatskiy M, Danilova N (2020). Cell Sheets from adipose tissue MSC induce healing of pressure ulcer and prevent fibrosis via trigger effects on granulation tissue growth and vascularization. Int J Mol Sci..

[109] Li Y, Wu M, Zhang Z, Xia J, Wang Z, Chen X (2019). Application of external force regulates the migration and differentiation of adipose-derived stem/progenitor cells by altering tissue stiffness. Tissue Eng Part A..

